# The epigenetic modifier *Fam208a* is required to maintain epiblast cell fitness

**DOI:** 10.1038/s41598-017-09490-w

**Published:** 2017-08-24

**Authors:** Shohag Bhargava, Brian Cox, Christiana Polydorou, Veronika Gresakova, Vladimir Korinek, Hynek Strnad, Radislav Sedlacek, Trevor Allan Epp, Kallayanee Chawengsaksophak

**Affiliations:** 1 Laboratory of Transgenic Models of Diseases, Division, BIOCEV, Institute of Molecular Genetics of the CAS, v.v.i., Vestec, Czech Republic; 2 Czech Centre for Phenogenomics, Division BIOCEV, Institute of Molecular Genetics of the CAS, v.v.i., Vestec, Czech Republic; 3 Laboratory of Cell and Developmental Biology, Institute of Molecular Genetics of the CAS, v.v.i., Krc, Czech Republic; 4 Laboratory of Genomics and Bioinformatics, Institute of Molecular Genetics of the CAS, v.v.i., Krc, Czech Republic; 5 Department of Physiology, Faculty of Medicine, University of Toronto, Ontario, Canada; 6 Faculty of Science, Charles University, Prague, Czech Republic

## Abstract

Gastrulation initiates with the formation of the primitive streak, during which, cells of the epiblast delaminate to form the mesoderm and definitive endoderm. At this stage, the pluripotent cell population of the epiblast undergoes very rapid proliferation and extensive epigenetic programming. Here we show that *Fam208a*, a new epigenetic modifier, is essential for early post-implantation development. We show that *Fam208a* mutation leads to impaired primitive streak elongation and delayed epithelial-to-mesenchymal transition. *Fam208a* mutant epiblasts had increased expression of p53 pathway genes as well as several pluripotency-associated long non-coding RNAs. *Fam208a* mutants exhibited an increase in p53-driven apoptosis and complete removal of p53 could partially rescue their gastrulation block. This data demonstrates a new *in vivo* function of *Fam208a* in maintaining epiblast fitness, establishing it as an important factor at the onset of gastrulation when cells are exiting pluripotency.

## Introduction

Gastrulation is a critical developmental process whereby the three germ layers (ectoderm, mesoderm and definitive endoderm; DE) are specified. Immediately post implantation and prior to gastrulation (E5.5 to E6.5), the mouse embryo dramatically changes in size and shape. The embryonic epiblast shows the highest proliferation rate (2–8 hours^1^,) in order to attain a critical cell number threshold^2,3^. Formation of the primitive streak (PS) at the posterior side of the embryo at E6.5 is the hallmark of gastrulation, and coincides with the completion of distal visceral endoderm (DVE) migration to the anterior side of the embryo to form the anterior visceral endoderm (AVE)^4^. As gastrulation progresses, the epiblast cells undergo an epithelial to mesenchymal transition (EMT) at the PS, giving rise to mesoderm and DE. Epiblast cells that do not ingress through the PS remain in the epiblast and give rise to ectodermal lineages such as the neurectoderm^5^. Gastrulation is also a period of dynamic epigenetic change, involving many different known epigenetic silencing factors, and likely others that are still to be discovered.

Several epigenetic silencing factors have been discovered in a dominant ENU mutagenesis screen in the mouse for modifiers of transgene variegation^6,7^. These were designated as modifiers of murine metastable epialleles or Momme. One group of genes identified in this screen are specifically involved in writing or reading repressive H3K9me3 marks; these are *MommeD9* (*Trim28/Kap1*), *MommeD13* (*Setdb1/Eset*), *MommeD33* (*Suv39h1*), and *MommeD*4*4* (*Trim33/ectodermin*)^7–9^. A new member to this list is *Fam*20*8a* (*MommeD6* and *MommeD20*^10^, which in human has recently been shown to be a core factor of a new epigenetic silencing complex comprising FAM208A, MPHOSPH8, PPHLN and SETDB1^11^. MPHOSPH8 through its chromodomain specifically binds H3K9me3, and SETDB1 catalyzes trimethylation of adjacent K9 residues. This complex, termed HUSH (human silencing hub) has been proposed to be important for heterochromatin spreading, as opposed to TRIM28-SETDB1 complexes which may be more important for *de novo* trimethylation when recruited to specific genomic sequences by members of the KRAB-zinc finger protein family^12^.

H3K9me3 is associated with tightly packed constitutive heterochromatin, typically found at pericentromeric and subtelomeric repeats, whereas facultative heterochromatin, typically found in silenced gene-encoding regions is associated with H3K9me2^13^. More recently it has been found that H3K9me3 also marks in embryonic stem cells, the poised state of master regulators of differentiation, allowing them to be acutely activated following inductive nodal-activin signalling^14^. These poised states are established by the action of Oct4, Sox2 and Nanog, which recruit Setdb1 to deposit the H3K9me3 mark^15^.

Loss of function mutations in mice of the above-mentioned H3K9me3-related genes, identified as modifiers of transgene variegation in the mouse, have been independently studied in an embryological context. All, except for the X-linked *Suv39h1*, result in early embryonic lethality; *Setdb1* null mice are lethal at the peri-implantation stage (E3.5–E5.5)^16^ while both *Trim33* and *Trim28* null mice fail to undergo gastrulation^17,18^. Previously, we reported that *MommeD6* and *MommeD20* homozygotes also die during the gastrulation stage. Here, we examine the mutant phenotype in more detail, characterizing their involvement in central morphogenetic events that occur during this stage, namely the establishment of anteriorio-posterior (A-P) patterning and EMT.

## Results

### Fam208a is widely expressed during early post implantation development

To investigate the role of *Fam208a* during post-implantation development, we first analysed its mRNA expression profile at embryonic stages preceding (E5.5), during (~E6.25 to 7.75) and following (E8.5) gastrulation. At E5.5 (egg cylinder; EC), *Fam208a* is specifically expressed only in the epiblast. At E6.5 (pre-streak; Pr-S), *Fam208a* expression extends into the extraembryonic ectoderm (ExE) and one day later E7.5 (early headfold; EHF), the expression is observed in embryonic ectoderm, allantois, amnion and chorion. From E8.5 to 9.5, *Fam208a* is ubiquitously expressed in the developing mouse embryo (Supplementary Fig. 1).

### Fam208a mutation leads to defective primitive streak elongation

**Figure 1:**
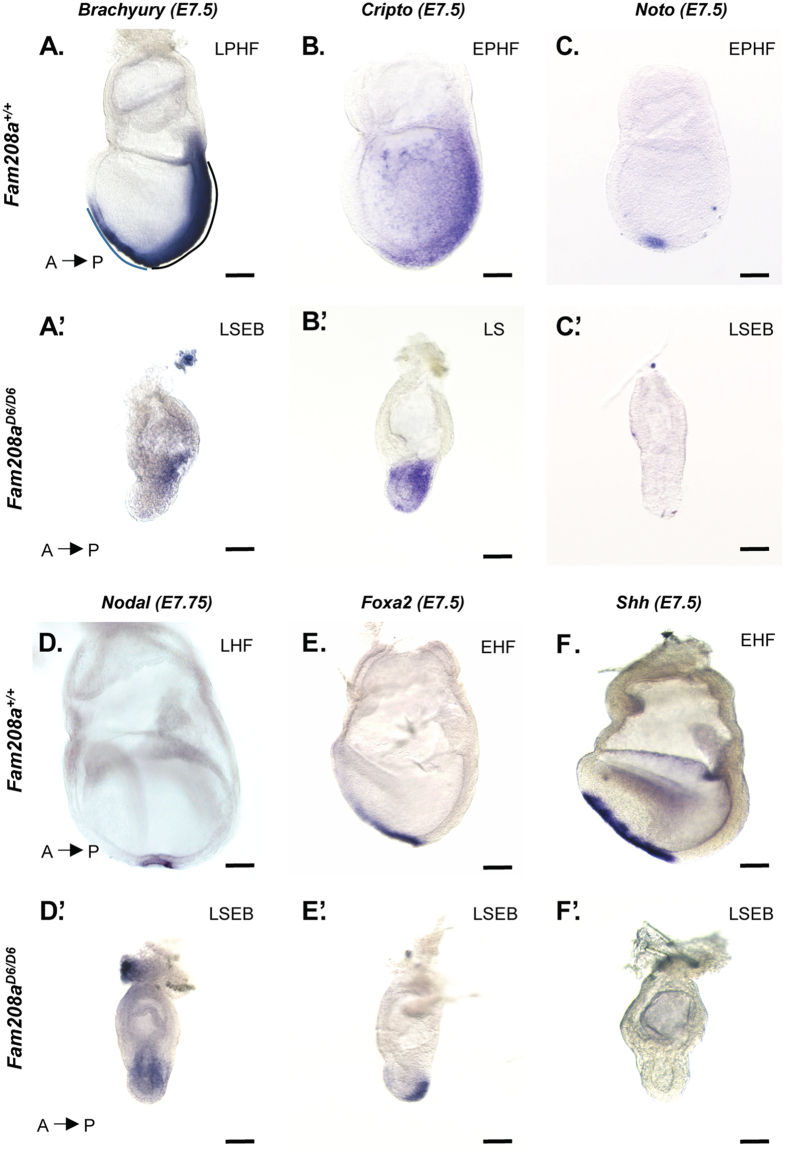
*Fam208a*^*D6/D6*^ mutants exhibit gastrulation failure defects. Whole mount *in situ* hybridization at E7.5-E7.75 of *Fam208a*^*D6/D6*^ mutants (**A’–F’**) and their wild-type littermate controls (**A–F**). The *Fam208a*^*D6/D6*^ mutant embryos at E7.5 are phenotypically distinguishable with severely retarded epiblast. (**A’**) In mutants, PS initiates but remains hardly 1/3^rd^ in its length with no distal and anterior expression as seen by Brachyury expression (pan-mesodermal marker). (**B’**) *Cripto*, a PS and nascent mesoderm marker is expressed slightly delayed in mutants. Together, they show arrested PS elongation. (**C’, D’** and **F’**) The expression of *Noto, Nodal* and *Shh* is undetectable in the node of the *Fam208a*^*D6/D6*^ mutant embryos with (**G’**) reduced anterior expression of AME marker *Foxa2*. Line indicates the length of the PS. Dashed line in black demarcates the length of the PS and the blue dashed line indicates the node and head process. Scale bar: 30 µm. PS, primitive streak, LPHF, Late pre-head fold; LSEB, Late streak, early allantoic bud; LS, Late-streak; EPHF, Early pre-head fold; LHF, Late Head fold; EHF, Early head fold; al, Allantois. Also, see Supplementary Figs 1–2.

From E6.5 (early streak; ES) onwards, *Fam208a*^*D6/D6*^ embryos were increasingly growth retarded. At later stages, the embryonic region became increasingly delayed while extraembryonic tissues continued to develop. At E7.5, we observed the expansion of the exocoelomic cavity with a small amniotic cavity which appears to form by the abutting of ExE onto itself, a lack of an amnion and an allantoic bud that was severely restricted in size (Fig. 1). The disparity between embryonic growth impairment and the relatively more advanced development of extraembryonic structures was consistent in both *Fam208a*^*D6/D6*^ and *Fam208a*^*D20/D20*^ mutants and therefore, we focused our subsequent studies on one of the mutant alleles, *Fam208a*^*D6/D6*^.

We first investigated the ExE development in *Fam208a*^*D6/D6*^ mutant embryos, by examining the expression of key marker genes such as *Cdx2*, *Elf5, Spc4*, and *Bmp4*, which have been shown to be important in ExE development and maintenance at the ES stage (E6.5)^19–22^. We observed comparable expression of *Elf5* (n = 4), *Spc4* (n = 2), *Cdx2* (n = 3) and *Bmp4* (n = 4) between *Fam208a*^*D6/D6*^ mutants and their littermate controls indicating that there is no major defect in ExE specification at E6.5 (Supplementary Fig. 2).

Gastrulation begins with the formation of the PS at E6.5. *Brachyury (T)* expression is widely used to mark the PS and axial mesoderm that migrates out of the PS but not the mesodermal wing^23^. While the expression of *T* in the E7.5 (late pre-headfold; LPHF) wild type embryo extends past the distal tip of the embryo and into the notochord precursor that extends anteriorly to the node (Fig. 1A), *T* expression in *Fam208a*^*D6/D6*^ embryos is restricted to the posterior of the embryo, extending distally about one-third of the length of the epiblast and never reached the distal tip. This suggests that while gastrulation is initiated, there is a failure to elongate the PS (*Fam208a*;^*D6/D6*^ n = 7; Fig. 1A’ and *Fam208a*;^*D20/D20*^ n = 3; Supplementary Fig. 3B). This was confirmed by examining *Cripto* expression. At the onset of gastrulation, *Cripto* is expressed in the PS and later, at E7.5 is also expressed in the mesodermal wing that extends rostrally^24^. *Cripto* seemed to be correctly expressed in *Fam208a*^*D6/D6*^ mutant embryos when compared to its wildtype littermate at E6.5 (n = 2, Supplementary Fig. 6). Further, *Cripto* expression in E7.5 *Fam208a*^*D6/D6*^ embryos which morphologically resembled E7.0 (midstreak; MS) is observed in the PS, but again the expression domain does not extend to the distal tip. *Cripto* expression extends further laterally and distally than that of *T*, marking also the migratory mesoderm that overlays the PS (n = 3; Fig. 1B’).

Next, we checked if there is correct specification of anterior mesendoderm (AME) and its main derivative the node, the organizer of the mouse gastrula. At E7.5, *Noto*, a marker of the node, is normally confined to the distal tip of the EC^25,26^ (n = 3; Fig. 1C). In *Fam208a*^*D6/D6*^ mutant embryos, *Noto* is absent (n = 3; Fig. 1C’), although expression does appear at E8.5–E9.5 (n = 2; Supplementary Fig. 4B’ and n = 3; Supplementary Fig. 4D’ respectively). Like *Noto*, *Nodal* expression is similarly confined to the node in wild-type littermate embryos at the E7.75 (late headfold; LHF) stage (n = 5; Fig. 1D), but in mutant littermates, *Nodal* expression reflects an earlier developmental stage, being strongly expressed in both the anterior and posterior proximal epiblast, with expression gradually reducing towards the distal epiblast and with no discernible presumptive node (Fig. 1D’).

We sought to confirm the lack of formation of the node and anterior PS derivatives in *Fam208a*^*D6/D6*^ mutant embryos by studying the expression of *Foxa2* and *Shh* during the late streak (LS) to EHF stage. While *Foxa2* and *Shh* share expression in the anterior definitive endoderm (ADE) and axial mesoderm^27–29^, *Foxa2* expression begins almost 24 hours prior to *Shh*^27,30^ at the ES stage, where it is localized to the posterior epiblast^31^ and delaminating mesoderm in the anterior PS. We observed absence of *Foxa2* expression (2/3) to very faint expression (1/3) in *Fam208a*^*D6/D6*^ mutant embryos when compared to its wildtype littermate at E6.5 (n = 3, Supplementary Fig. 6). *Shh* expression is first detected at the early allantoic bud (EB) stage in the midline mesoderm of the head process. Later, at the late streak early allantoic bud (LSEB) stage, *Shh* expression is initiated in the node, the notochord, and later in the DE^27^ – overlapping expression domains with *Foxa2*. In the littermate controls at E7.5 (EHF), *Foxa2* expression is consistent with this stage of development, being in the node, ADE and axial mesoderm. In *Fam208a*^*D6/D6*^ mutants however, *Foxa2* expression is delayed and is seen in the posterior epiblast and mesoderm similar to that of ES stage (n = 4, Fig. 1E’). This developmental delay is further confirmed by the complete absence of *Shh* at E7.5 (n = 5; Fig. 1F’) and E8.5 (n = 2; Supplementary Fig. 3C’) while *Foxa2* was expressed in the anterior midline of both *Fam208a*^*D6/D6*^ as well as littermate controls at E7.5 (EHF). There is no change in *Foxa2* expression in *Fam208a*^*D6/D6*^ embryos at E8.5, when the epiblast appeared as a EC with no headfold initiation, yet with a discernible allantoic bud (*Fam208a*^*D6/D6*^, n = 2; Supplementary Fig. 3D’ and *Fam208a*;^*D20/D20*^ n = 2; Supplementary Fig. 3D”). Despites defective elongation and inability to give rise to notochordal cells, we detect Noto positive cells an indicator of node activity in *Fam208a* mutants at E8.5 (Supplementary Fig. 4B’). Collectively, we conclude that embryos with *Fam208a* mutation can develop with no overt morphological changes to the ES stage and can initiate gastrulation after which development becomes increasing delayed and fails to progress beyond the EHF stage.

### Fam208a is important for epithelial-to-mesenchymal transition at the onset-of gastrulation

**Figure 2:**
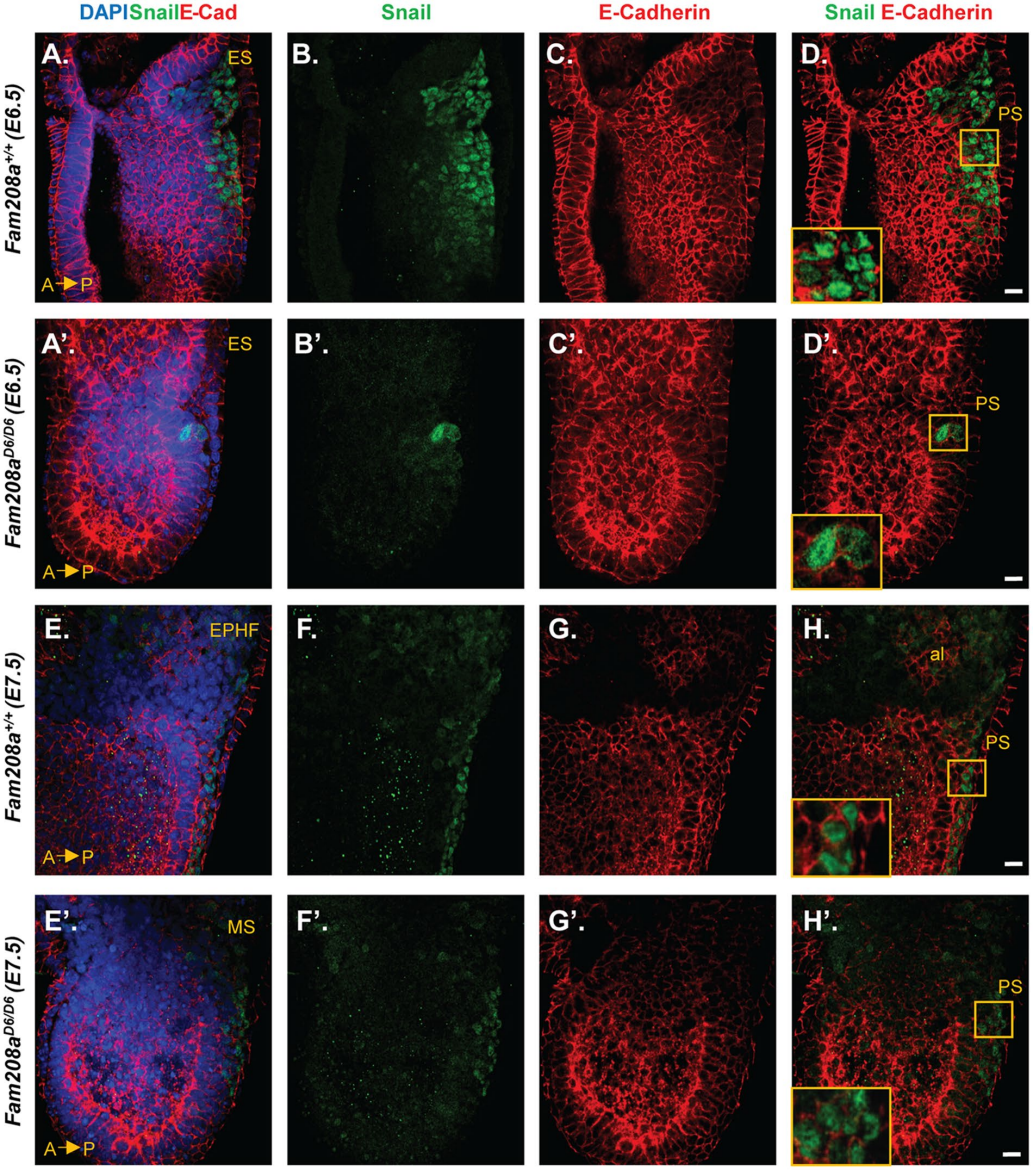
*Fam208a*^*D6/D6*^ mutants exhibit significantly delayed epithelial-to-mesenchymal transition during gastrulation. Whole mount immunofluorescence of mutant *Fam208a*^*D6/D6*^ embryos (E6.5: **A’–D’** and E7.5: **E’–H’**) and their wildtype littermates (E6.5: **A–D** and E7.5: **E–H**). (**D’**) Confocal images show only a very few Snail-expressing (mesodermal marker, green) cells within the PS with failure to down-regulate E-cadherin (epiblast and endodermal marker, red) at E6.5. Snail expression increases along the elongated PS by E7.5 but gets arrested halfway. The boxed region to the bottom left is of 4-fold magnification. Scale bar: 30 µm. ES, Early streak; EPHF, Early pre-head fold; MS, Mid-streak; PS, Primitive streak; Al, allantois.

During gastrulation, epiblast cells undergo EMT, migrate and ingress through the PS and later emerge as differentiated cells to form the mesoderm, a new layer between the epiblast and the overlying visceral endoderm (VE)^32,33^. We investigated the expression of two markers of EMT, namely E-cadherin and Snail. Prior to gastrulation, E-cadherin, encoded by the *Cdh1* gene, is expressed in the epiblast and endoderm^34^ and is downregulated in those epiblast progenitor cells that delaminate and undergo EMT at the PS, and is no longer expressed in the nascent mesoderm^33^. In contrast, *Snail* is first detected within the PS and in the migratory mesodermal wings^35^. *Snail* is a transcriptional repressor that acts downstream of Fgf signaling to repress *Cdh1* gene expression^33,36,37^. In *Fam208a*^*D6/D6*^ mutant embryos, we analysed the protein level of E-Cadherin and Snail by whole-mount immunostaining, both at the onset (E6.5; ES) and during the progression of gastrulation (E7.5, Early pre-head fold, EPHF). At E6.5 (ES), there are either no (n = 1/3) or just a couple of *Snail*-expressing epiblast cells (n = 2/3) with no significant reduction in E-cadherin expression (Fig. 2A’–D’) when compared to wildtype littermates (Fig. 2A–D). In *Fam208a*^*D6/D6*^ mutants at E7.5, which morphologically resemble E7 (MS), there were an increased number of Snail-expressing cells marking the nascent PS, which remained at the MS stage and never extended to the distal tip, as observed in control littermates (n = 5/5; Fig. 2F’–H’). Therefore, we conclude that *Fam208a*^*D6/D6*^ mutants can initiate EMT but are unable to sustain progression, leading to a shortened PS.

### Fam208a mutant embryos exhibit an alteration in anterior-posterior patterning

**Figure 3:**
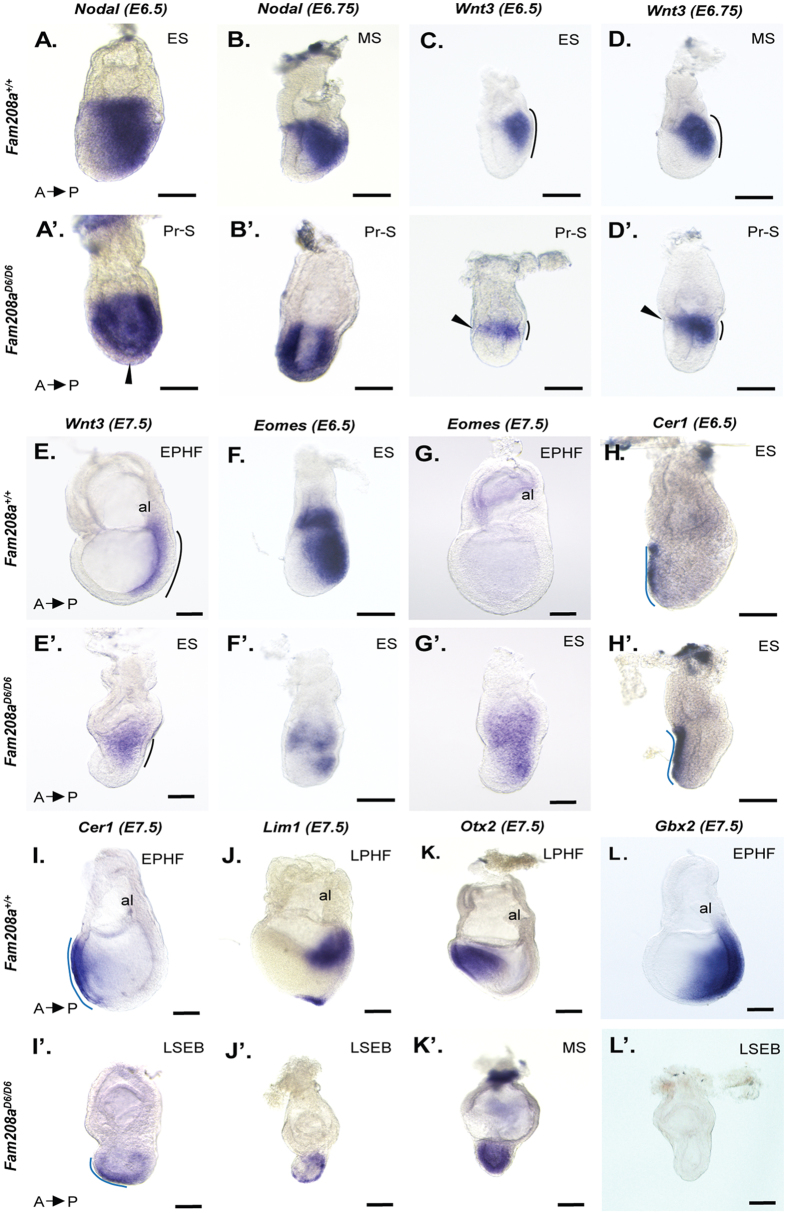
Gene marker expression in *Fam208a*^*D6/D6*^ mutant embryos. Whole mount *in situ* hybridization at E6.5-E7.5 of *Fam208a*^*D6/D6*^ mutants (**A’–L’**) and their wild-type littermates (A–L). (**A’–D’**) Posterior epiblast markers *Wnt3* and *Nodal* fail to be completely down-regulated anteriorly in mutant embryos at E6.5–6.75. (F’) *Eomes* (key-regulator of EMT and inducer of mesoderm) is down-regulated in E6.5 mutant embryos. (**I**’) complete absence of *Gbx2* (posterior neuroectoderm; hindbrain marker) (**J**’) with expanded (both anterior and posterior) *Otx2* (anterior forebrain marker) expression domain in E7.5 mutants. (**H**’) slight *Lim1* (anterior PS marker) expression is seen at E7.5 in mutants when compared to wild type. (**K’,L**’) Note that AVE migrates correctly in mutants at E6.5. At E7.5, *Cer1* is expressed in the ADE overlaying future head formation, while in mutants *Cer1* expression is reduced and remains in the distal epiblast. Dashed line in black indicates the length of the primitive streak and in blue indicates the length of the AVE. Scale bar: 30 µm. ES, Early streak; MS, Mid-streak; Pr-S, Pre-streak; EPHF, Early pre-head fold; LSEB, Late streak, early allantoic bud; LPHF, Late pre-head fold; al, allantois.

Because of the delay in gastrulation progression in *Fam208a* mutants (both *Fam208*^*D6/D6*^ and *Fam208a*^*D20/D20*^), we sought to analyse several regulatory genes expressed within the *Fam208a*^*D6/D6*^ epiblast from E6.5–7.5. First, we examined the expression of *Nodal*. In normal embryos, at E5.5 (EC stage), *Nodal* is expressed throughout the VE and epiblast^38^. As gastrulation progresses, *Nodal* is rapidly downregulated in the VE and anterior ectoderm and becomes concentrated in the posterior ectoderm, which is indeed observed in our E6.5 (ES) littermate embryos (Fig. 3A). In contrast, in E6.5-E6.75 *Fam208a*^*D6/D6*^ embryos, although downregulated in the VE (arrowhead), *Nodal* remains expressed in the anterior epiblast (n = 4; Fig. 3A’,B’).

Because of this failure to downregulate anterior *Nodal* expression in *Fam208a*^*D6/D6*^ embryos, we investigated expression of *Wnt3* and *Eomesodermin (Eomes)*, two downstream targets of Nodal signaling^38^. In E6.5 (ES) *Fam208a*^*D6/D6*^ mutant embryos, *Wnt3* is localized to the posterior epiblast, albeit with reduced expression and extending more anteriorly (arrowhead Fig. 3D’) to that of littermate controls (n = 2; Fig. 3C’). By E6.75 (n = 2; Fig. 3D’), *Wnt3* expression in *Fam208a*^*D6/D6*^ embryos has increased, but its expression domain extends only one third the length of the epiblast, instead of the two-thirds seen in the control littermates, and remains unchanged at E7.5 (n = 2; Fig. 3E’).

Next, we studied the expression of *Eomes* because it is induced by Nodal in the ExE and in the posterior epiblast prior to PS formation^38^ and because its function is crucial for mesoderm formation^39^. During gastrulation, *Eomes* is expressed in the PS and nascent mesoderm, and later becomes confined to the anterior PS, where it abruptly disappears prior to node formation^40–42^. In *Fam208a*^*D6/D6*^ mutants at E6.5 (ES), *Eomes* is significantly down-regulated in both extra embryonic tissues and in the posterior epiblast (n = 5; Fig. 3F’). At E7.5, *Fam208a*^*D6/D6*^ embryos morphologically resemble E6.5 (LSEB) and *Eomes* expression slightly increases, but still remains restricted to the ExE and the PS (n = 2; Fig. 3G’). In contrast, in the littermate control at E7.5 (EPHF), extraembryonic expression is seen only in the chorion as embryonic expression has already disappeared.

To evaluate whether failure to downregulate *Nodal* expression in *Fam208a*^*D6/D6*^ mutants is simply due to a developmental delay or whether lack of *Fam208a* alters the regulatory network of Nodal signalling, we examined the expression profile of several known genes involved in A-P patterning. *Cer1*, a Nodal antagonist, is an important marker of the AVE^43^. In the ES stage, *Cer1* expression is detected in the AVE extending towards the embryonic distal tip. By MS stage, *Cer1* is detected in the VE and in DE emerging from the node. *Cer1* expression in DE later disappears, remaining only in the midline (AME) underlying the future head formation^44–46^. In E6.5 (ES) *Fam208a*^*D6/D6*^ mutants *Cer1* is correctly expressed in the AVE and is comparable to littermate controls (n = 3; Fig. 3H’). Strikingly, the *Cer1*-expression in E7.5 *Fam208a*^*D6/D6*^ embryos, which morphologically resembled the LSEB stage, either was absent (n = 3/7) or was confined to the endoderm at the distal tip, potentially marking the precursors of DE (n = 4/7; Fig. 3I’). We hypothesized that *Cer1* positive cells in *Fam208a*^*D6/D6*^ embryos at the ESEB stage were endodermal cells and not AME cells because of the lack of *Foxa2* (another AME marker) expression in *Fam208a*^*D6/D6*^ embryos (Fig. 1E’). To further confirm the lack of AME, we studied the expression of *Lim1*. In ES-LS stages, *Lim1* expression is confined to the AVE and to the PS. By LS to EHF (E7.5–7.75), *Lim1* is expressed in the mesendoderm (node and notochord) and lateral plate mesoderm^47,48^. *Lim1* expression in *Fam208a*^*D6/D6*^ mutants at E7.5 (LSEB) showed patchy expression in the VE and in the PS, similar to the *Lim1* expression pattern reported for normal embryos at ES stage (n = 4; Fig. 3J’). *Otx2*, an anterior forebrain/midbrain marker, is widely expressed in the epiblast from the PrS-ES stage. During MS-LS, *Otx2* expression is progressively reduced in the posterior epiblast and then becomes limited to the anterior half of the embryo^49,50^. At E6.5, *Otx2* is robustly expressed within the entire epiblast of *Fam208a*^*D6/D6*^ embryos, while in littermate controls, its expression domain has already shifted anteriorly (n = 2; Supplementary Fig. 6). *Otx2* expression in E7.5 *Fam208a*^*D6/D6*^ mutants (LS) still remains located throughout the epiblast, and is reminiscent to that of normal embryos at the PrS-ES stage (n = 2; Fig. 3K’). In the LS-EHF stage, *Gbx2*, a posterior neuroectoderm marker, is widely expressed in the posterior part of the embryo and is excluded from the headfold^51^. *Gbx2* expression is completely absent in E7.5 *Fam208a*^*D6/D6*^ mutant embryos (n = 5; Fig. 3L’).

### Determination of overall cell number and proliferation rates in Fam208a mutants

**Figure 4:**
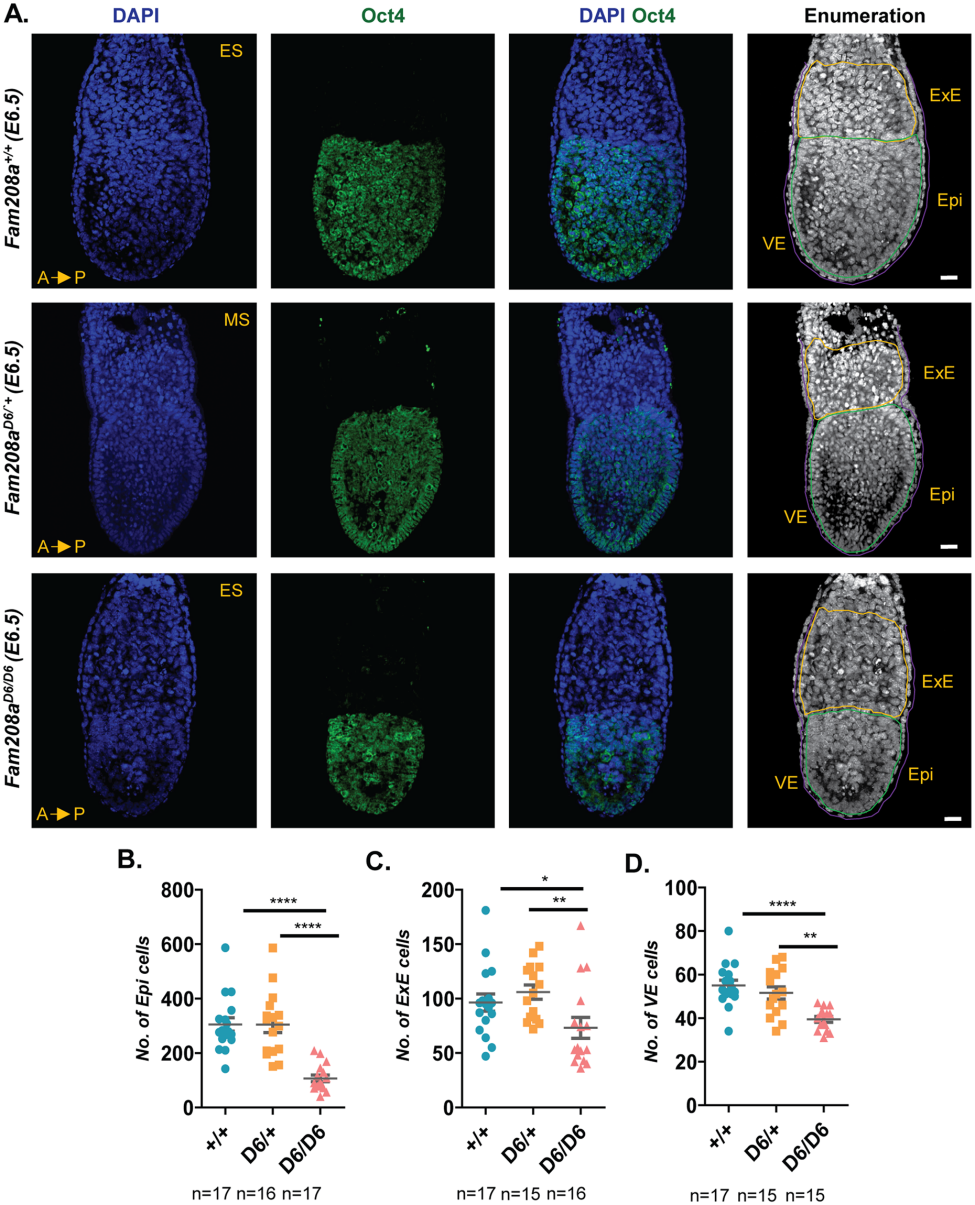
*Fam208a*^*D6/D6*^ mutants have reduced cell numbers. Whole mount immunofluorescence of mutant *Fam208a*^*D6/D6*^ mutant embryos and their wildtype littermate controls at E6.5. (**A**) Confocal images show that mutant embryos have a smaller epiblast as seen by the smaller expression domain of Oct4 (epiblast marker, green). Quantification of (**B**) epiblast, (**C**) ExE and (**D**) VE cell numbers. All results are mean ± SEM from 17 (*Fam208a*^+/+^), 15 (*Fam208a*^*D6*/+^) and 15 (*Fam208a*^*D6/D6*^) embryos. *p < 0.05, ****p < 0.0001 Scale bar: 50 µm. ES, Early streak; MS, Mid-streak; ExE, Extra-embryonic ectoderm; Epi, Epiblast; VE, Visceral endoderm.

**Figure 5:**
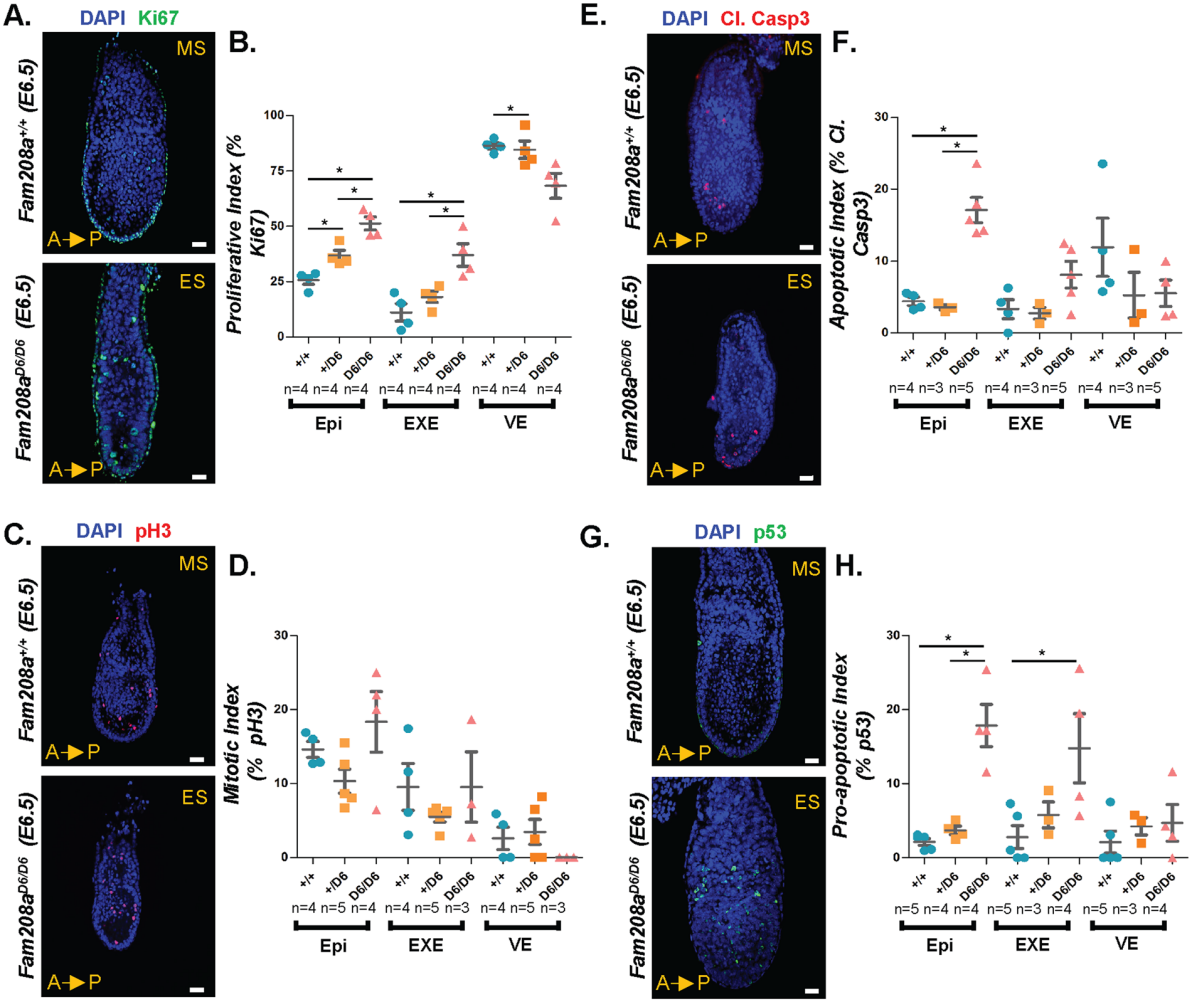
*Fam208a*^*D6/D6*^ mutants exhibit altered proliferation and increased p53-mediated apoptosis. (**A,B**) Confocal images of mutant embryos with significantly increased Ki67-positive cells (green) in Epi and ExE; *p < 0.05. (**C,D**) The mutant embryos show no significant change in pH3 (red) expression, a measure of mitotic index. (E-F) *Fam208a*^*D6/D6*^ mutant embryos have significantly increased apoptosis as shown by Cleaved Caspase3 positive cells (Cl. Casp3; Red) particularly in the epiblast, *p < 0.05 (**G,H**) also with pronounced increase in p53 level primarily in the epiblast and in part of the ExE region adjacent to the epiblast, *p < 0.05. All results are calculated as mean ± SEM from at least two different litters. The number of embryos analysed for each marker are indicated.

To investigate if the overt developmental phenotype of *Fam208a*^*D6/D6*^ mutant embryos was due to an overall decrease in cell number, we first quantified the number of cells in three distinct regions, namely the epiblast, ExE and VE at E6.5. We found a significant reduction in cell number for all three regions in *Fam208a*^*D6/D6*^ mutant embryos when compared to littermate controls (epiblast, n = 17; ExE, n = 15; VE, n = 15; Fig. 4B–D). Next, we performed immunofluorescence staining using the pan-proliferation marker Ki67 to determine the proliferative index in the epiblast, ExE and VE. We found a significant gene-dosage dependent increase in the proliferative index compared to its littermate controls (n = 4; Fig. 5A,B). To examine further this defect, we also checked the M phase marker, phospho-H3 (Ser 28, M-phase marker^52^ but found no significant change in the mitotic index in *Fam208a*^*D6/D6*^ mutant embryos (n = 3 embryos; Fig. 5C,D). This suggests that the increased percentage of cells that have entered the cell cycle (Ki67) are arrested or delayed in a phase of the cycle other than M-phase (phospho-H3). These findings cannot account for the diminished size of *Fam208a*^*D6/D6*^ mutant embryos and therefore, we shifted our focus to examining the rate of apoptosis.

### Fam208a mutants exhibit increased apoptosis and are partially rescued by p53 mutation

The epiblast, with its very high rates of cell proliferation, is under constant replicative stress and is particularly sensitive to genotoxic stresses^53^. In response to genotoxic stress, epiblast cells will normally undergo rapid apoptosis^54^. We investigated whether an increase in apoptosis was leading to impaired epiblast growth using cleaved caspase-3 imunofluorescence as a measure of the apoptotic index. *Fam208a*^*D6/D6*^ mutant epiblasts had a significant increase in the number of cleaved caspase-3 positive cells at E6.5. This increase was specific to the epiblast, and was not observed in either the ExE or VE (n = 4; Fig. 5E,F).

A p53-dependent apoptosis-mediated mechanism increases embryo fitness by removing mutated or damaged epiblast cells during early post-implantation development, allowing the selective clonal expansion of healthy cells^53^. To determine whether p53 activation was associated with the observed increase in apoptosis in E6.5 *Fam208a*^*D6/D6*^ embryos, we investigated the expression of p53 using immunofluorescence. We found a significant increase in p53 expression in the epiblast as well as within the ExE-epiblast junction in *Fam208a*^*D6/D6*^ mutants (n = 4; Fig. 5G,H).

**Figure 6:**
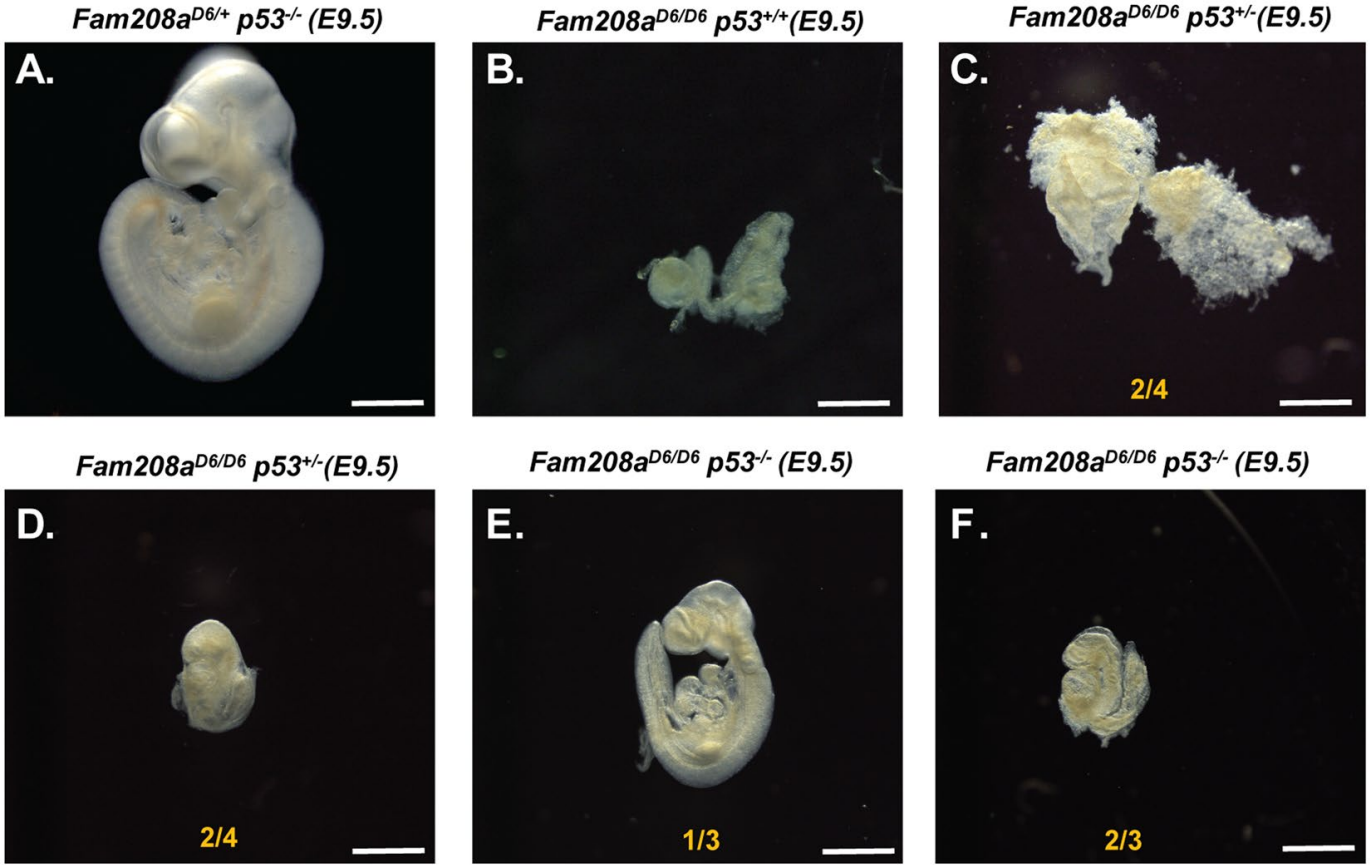
Partial rescue of *Fam208a*^*D6/D6*^ mutant gastrulation block upon p53 removal. Gross morphology of E9.5 embryos obtained from *Fam208a*;^+*/D6*^ *p53*^+/−^ intercrosses. (**A**) Representative bright-field image of normally developing *Fam208a*;^+/+^ *p53*^−/−^ control embryos, (**B**) no rescue of the *Fam208a*^*D6/D6*^ phenotype is observed as a result of the introduced mixed background (FVB/N and C57BL/6 J), however variable rescue of the *Fam208a*^*D6/D6*^ phenotype is observed in embryos with p53^+/−^ (**C,D**) or p53^−/−^ (**E,F**) genotypes. In all cases, representative embryos of each genotype are shown. Scale bar: 100 µm.

Several gene knock-out models exhibiting gastrulation failure with p53 dependent apoptosis can be rescued by crossing to *p53* mutant mice^55–59^. To investigate whether the gastrulation block seen in *Fam208a*^*D6/D6*^ mutants could be similarly rescued in a *p53*^*−/−*^ background, we inter-crossed double heterozygous *Fam208a;*^*+/D6*^ *p53*^*+/−*^ mice and dissected at E9.5, a time point when *Fam208a*^*D6/D6*^ mutant embryos are severely retarded and are morphologically similar to the E6.75–7.0 stage. Indeed, we observed a partial rescue, *Fam208a:*^*D6/D6*^*: p53*^*−/−*^ double nullizygous embryos were alive judging by a beating heart at the time of dissection. They reached developmental milestones associated with E8.5–9.0 with several developmental abnormalities, including neural tube closure defects (open mid and hind-brain), an abnormal and enlarged pericardium, and irregular/smaller somites (n = 3; Fig. 6E,F and Supplementary Fig. 5). There was also detectable rescue seen in half of *Fam208a*^*D6/D6*^ embryos in a *p53*^*+/−*^ heterozygous background (n = 2/4; Fig. 6D); for the other half only an empty yolk sac was retrieved (n = 2/4; Fig. 6C). These results clearly indicate that the developmental phenotype seen in *Fam208a* mutant embryos is due to a p53-dosage mediated increase in the rate of apoptosis.

### Transcription profiling in Fam208a mutant epiblasts

An expression microarray was performed using total RNA isolated from single dissected epiblasts at E6.25. The experiment consisted of four samples from each genotype (*Fam208a*^*D6/D6*^, *Fam208a*^*+/D6*^ and *Fam208a*^*+/+*^), equally represented by gender. As the *MommeD6* line was originally identified as a semi-dominant suppressor of transgene variegation, we included heterozygous samples in our analysis in an attempt to identify any dosage-dependant changes in transcript abundance.

**Figure 7:**
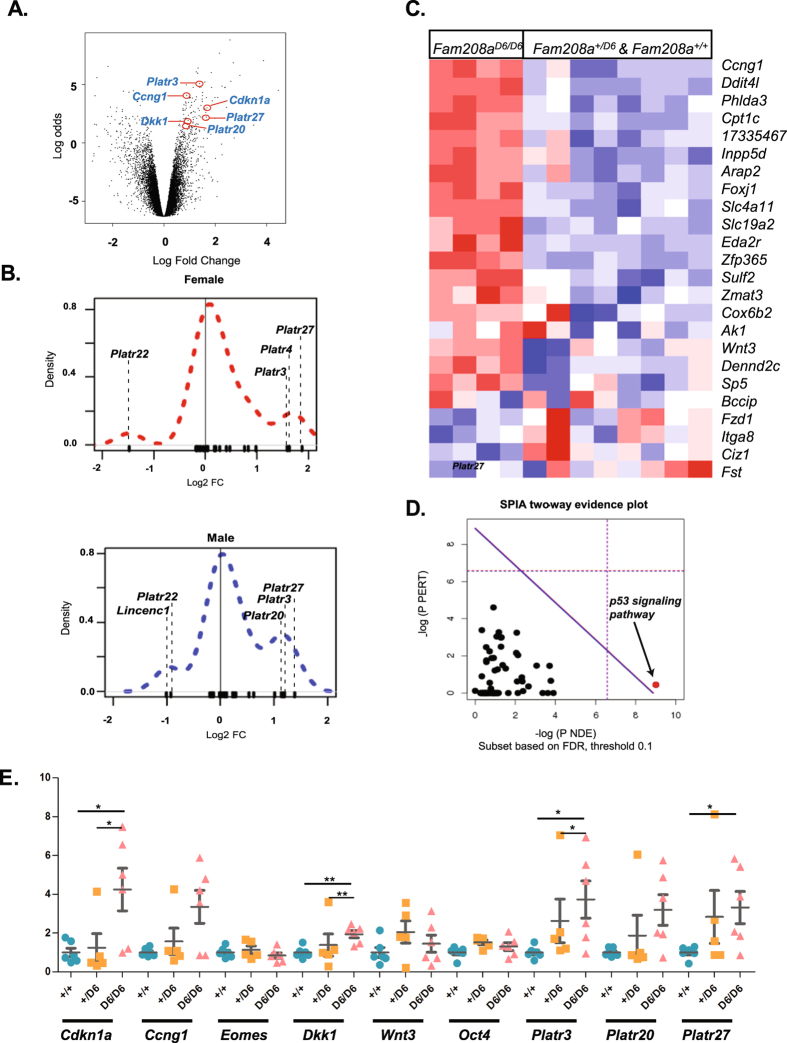
Increased p53 signaling and deregulation of pluripotency-associated transcripts in *Fam208a*^*D6/D6*^ epiblasts. (**A**) Volcano plot of *Fam208a*^*D6/D6*^ contrast with *Fam208a*^+/+^ indicating position of selected genes selected for validation by qPCR. (**B**) density plots showing position of *Platr* genes in all genes differentially expressed between *Fam208a*^*D6/D6*^ and *Fam208a*^+/+^ epiblasts with data segregated according to sex (*n* = 2 each) (**C**) heat map showing differential expression of a *p53*-bound, *p53/p73*-regulated gene set defined by [7] (**D**) statistical overrepresentation of the p53 signaling pathway using SPIA analysis (**E**) single epiblast qPCR verification at E6.25 for gene expression normalized to Gapdh with mutant values represented as a fold change relative to wildtype littermate (average expression converted to 1). Expression measurements were carried out in duplicates per embryo (number of embryos; +/+, n = 6; +/D6, n = 4, D6/D6, n = 6). All results are calculated as mean ± SEM from at least two different litters.

Using signalling pathway impact analysis (SPIA)^60^ against the KEGG database, we saw significant enrichment of the p53 signalling pathway (mmu04115, *p* = 0.0023) (Fig. 7D). This enrichment is readily apparent in a related 24 member gene set defined recently^61^, comprising the p53-bound (50 kb from TSS) subset of genes significantly downregulated in response to combined p53/p73 depletion in mouse embryoid bodies (Fig. 7C). For this p53/p73 dataset not only was there significant enrichment in homozygotes (comp. probability <0.001, NE_k_ q <0.001, Supplementary Table. 1), but also in heterozygotes (comp. probability = 0.005, NE_k_ q = 0.094).

Also revealed in homozygotes, were increased levels of several transcripts related to embryogenesis. These include *Dkk1* (14.2 fold, q = 1.1 × 10^−5^), *Gsc* (4.2 fold, q = 1.5 × 10^−4^), *Cfc1* (*Cryptic*) (5.9 fold, q = 0.0018), and *Nodal* (2.5 fold, q = 0.0012) indicating that altered expression of important patterning genes is already present at E6.25, a stage when mutant embryos are morphologically indistinguishable from wild-type. For *Dkk1* this increase was confirmed by qRT-PCR (Fig. 7E). We also observed a significant overrepresentation of KRAB protein-containing (IPR001909) genes (comp. probability = 0.001, NE_k_.q = 0.071), which is consistent with the results from *FAM208A* knockdown experiments using human cell lines^11^.

Functional set enrichment was also found for a previously defined *Oct4* coexpression-based pluripotency module (comp. probability = 0.005, NE_k_ q = 0.006)^62^ suggesting that mutant epiblast cells may have a delayed transition from a naïve to a primed pluripotency state, or alternatively delayed exit from pluripotency. The long non-coding RNA fraction from this gene module, termed *Platr* (pluripotency associated transcript), was particularly enriched (comp. probability <0.001, NE_k_ q = 0.003) with a similar distribution profile between males and females (Fig. 7B). For *Platr3, Platr20* and *Platr*27, this upregulation was confirmed by qRT-PCR (Fig. 7E). Notably, the *Platr* gene set was also enriched in heterozygotes (comp. probability <0.044, NE_k_ q = 0.347).

## Discussion

Extensive epigenetic changes occur at the onset of gastrulation as cells exit pluripotency and become committed to the different embryonic germ lineages. Here, we show that the epigenetic repressor *Fam208a* is vitally important at this stage, with *Fam208a*^*D6/D6*^ mutant embryos exhibiting profound developmental delay beginning by E6.5.

The establishment of the anterior-posterior axis occurs during gastrulation and is coordinated with embryonic growth. In *Fam208a*^*D6/D6*^ embryos we observed significantly impaired growth in the epiblast at E6.5, a time corresponding with the onset of gastrulation and the establishment of A-P patterning. Strongly supporting the interpretation of developmental delay is the eventual appearance of node activity at E8.5 (Supplementary Fig. 4). However, we did observe discrepencies that cannot be readily attributed to developmental delay. First is the increasingly desynchronized growth of extraembryonic tissue compared to embryonic tissue and second, is a failure to downregulate anterior *Nodal* expression.

Normally expression of *Lefty1* and *Cer1* in the DVE inhibits *Nodal* expression, resulting in a proximal-distal Nodal gradient. This gradient rotates to become the A-P axis, whereby the DVE moves anteriorly to form the AVE, and the proximal epiblast will move posteriorly^63–66^. In E6.5 *Fam208a*^*D6/D6*^ embryos this rotation has occurred, with the AVE clearly visible as an anterior *Cer1*-expression domain, but without the expected effect of downregulating anterior *Nodal* expression (Fig. 3). This is not the case for *Wnt3* expression, a source of inhibitory signalling to AVE, which rotates normally to the posterior epiblast. Similar to our *Fam208a*^*D6/D6*^ embryos, *Drap1* and *Lefty2* knockouts also have gastrulation failure with excessive *Nodal* signaling, but are able to correctly specify the AVE^67,68^. A possible reason for the rescue of AVE development is the highly increased expression of *Dkk1* in E6.5 *Fam208a*^*D6/D6*^ epiblasts. Dkk1 is an attractive signal for AVE migration and exogenous administration of Dkk1 can rescue AVE migration defects caused by inhibition of proliferation^69^, suggesting some plasticity in the coordination of A-P axis formation with embryonic growth.

The gastrulation defective *Fam208a*^*D6/D6*^ mutants exhibited upregulation of p53-signature genes and increased p53 protein stability. Critically, *Fam208a*^*D6/D6*^ mutants when crossed into a p53 null background, showed a rescue of the gastrulation phenotype. The rescued embryos, with their kinked neural tube, cardiac defects and failure of anterior neural tube closure, resembled p53/p63/p73 triple knockout chimaeras in which the phenotypes were attributed to an impairment in mesendodermal specification with a corresponding proclivity to assume a neurectoderm fate^61^.

The epiblast has been shown to contain an amplified p53 signaling response and high cytoplasmic priming towards apoptosis. This heightened vigilance protects against the accumulation of mutations, at a formative period when rapid cell cycling and a relatively open chromatin conformation can make them more sensitive to genomic insults. Indeed the period between E5.5 and E7.5 comprises a sensitive developmental window during which the deletion of many genes important for genome integrity is lethal^70–74^. Possible reasons for an increase in p53-dependent apoptosis in *Fam208a*^*D6/D6*^ embryos are increased genomic instability, for example due to impaired repression of endogenous retroviruses and satellite repeats, or it could be the result of stabilization of the p53 protein itself. *Fam208a*^*D6/D6*^ embryos at E6.5 exhibited a gene dosage-dependent increase in the percentage of cells labeled with the proliferative marker Ki67. This increased index of proliferation is possibly a compensatory response to cellular attrition through p53-mediated apoptosis and cell cycle arrest. Similar models of compensatory epiblast growth have been described^2,3^.

*Fam208a* (then termed *D1*4*Abb1e*) was first proposed as a pluripotency-related gene based on its clustering within an *Oct4* co-expression module and demonstration of *Oct4* occupancy of its promoter^75^. Our expression profiles of *Fam208a*^*D6/D6*^ mutant epiblasts show gene set enrichment for an *Oct4* co-expression module^62^, and in particular in the nuclear long non-coding subset of this co-expression module, which have been termed pluripotency-associated transcripts (*Platr*). Indeed *Platr3*, -*4*, -*20* and -27 were all within the top 95 up-regulated genes (q-value < 0.05 and fold change >2). However, not all members of this module were similarly affected, and *Platr22* actually decreased in expression. None of the dysregulated lncRNAs in *Fam208a* mutants have been previously ascribed a functional role in maintaining embryonic stem cell pluripotency, although direct functional roles have so far been described for *Platr11* (*linc1405*;^76^) *Platr14*^62^ and *Platr18* (*Lincenc1*;^77^). It is also possible that these lncRNAs are markers for more global, repressive epigenetic changes associated with pluripotency exit. If so, then silencing of *Platr3*, -*4*, -*20* and -*27* may be especially sensitive to *Fam208a*-dependent expansion of H3K9me3 domains whereas other *Platr* genes may rely more on other mechanisms of epigenetic silencing.

In summary, our results show an important role for *Fam208a* in maintaining epiblast fitness, and in its absence, embryos are subject to loss via p53-mediated apoptosis. Rescuing the phenotype by mutating *p53* brings the question of whether mutation or misexpression of *Fam208a* can similarly effect fitness of other cell populations, and whether such fitness defects are similarly “rescued“ by loss of the tumor suppressor *p53*, leading to cancer.

## Materials and Methods

### Ethics statement

Housing of mice and *in vivo* experiments were performed in compliance with the European Communities Council Directive of 24 November 1986 (86/609/EEC) and national and institutional guidelines. Animal care and killing mice by cervical dislocation were approved by the Animal Care Committee of the Institute of Molecular Genetics (Ethic approval ID 14/2015).

### Mouse lines and embryo collection

The two mutant strains of *Fam208a*, namely *MommeD6* (L130P) and *MommeD20* (IVS1 + 2 C > T), were maintained by inbreeding on the FVB/NJ background and have been described previously^10^. *Trp53*^*tm1Tyj*^ mice^78^ were obtained from the Jackson Laboratories (Bar Harbor, USA) and were maintained on a C57BL/6J background. All mice were kept under specific pathogen free conditions according to Federation of European Laboratory Animal Science Associations (FELASA) recommendations and all procedures were in strict accordance with local Animal Ethics Committee regulations. Embryos were harvested from timed matings of *Fam208a*^*D6/+*^ or *Fam208a*^*D20/+*^ intercrosses, with noon of the day on which the plug was observed designated embryonic day E0.5. For more accurate staging, we followed the revised Theiler staging of mouse development before organogenesis^79^.

### Genotyping

For *MommeD6* or *MommeD20* strains, PCR products amplified from the whole embryo or the Reichert’s membrane were used for genotyping by Sanger sequencing as described^10^. For gender PCR, Ube primers and conditions were used^80^ and for *Trp53*^*tm1Tyj*^ genotyping, we followed distributor’s protocol (Jackson Laboratory, Bar Harbor, USA).

### Whole-mount *in situ* hybridization and histology

Embryos were dissected from time mating females into cold PBS containing 10% FBS and were fixed overnight in 4% paraformaldehyde in PBS containing 0.1% Tween-20 at 4 °C (PBT). Single-color whole mount *in situ* hybridization was carried out as described^81^. RNA probes were either labelled with digoxygenin (DIG - Roche Diagnostics, Germany) or FITC (Roche Diagnostics, Germany). The riboprobe template for *Fam208a* was prepared using the primers Fwd- ACCACTGGAGAAGCCTGAGA and Rev- GGAATCTTCCTGCTGCACTC and templates for *T, Nodal, Cer1, Foxa2, Shh, Noto, Wnt3, Eomes, Gbx2, Lim1*, and *Otx2* were obtained from Prof. Janet Rossant and were used previously^82^. After post-fixing overnight in 4% paraformaldehyde, embryos were imaged using an inverted microscope (SteREO Discovery V12, Zeiss). Selected embryos were then washed 5–6 times in PBT, embedded in agarose and then embedded in paraffin for sectioning at 3 µm for haematoxylin and eosin (H&E) staining. Sections were imaged using a Zeiss Imager.Z2 equipped with objective N-Achroplan 40x/0.65 M27 and ZEN Software for image acquisition.

### Whole mount Immunofluorescence

Dissected embryos were fixed in 2% paraformaldehyde in PBT at room temperature for 20 mins and washed twice in PBT. Embryos were permeabilized in 0.1 M glycine/0.1% Triton X-100 for 12 mins (E6.5) or 15 mins (E7.5) at room temperature and washed twice in PBT. The embryos were blocked in 10% FBS/1%BSA in PBT (blocking buffer) at room temperature for 3 hrs. For primary mouse antibodies, the embryos were further blocked using the mouse MOM IgG kit (Vector Laboratories) according to the manufacturer’s instructions. Embryos were incubated overnight with primary antibodies diluted in blocking buffer and the following day, were incubated further in primary antibodies for 2 hours at room temperature, washed three times in PBT for 10 minutes each and incubated in secondary antibodies diluted in blocking buffer for 3 hours at room temperature. The embryos were washed three times with PBT, stained with DAPI (nuclei), mounted, and confocal imaged using a Leica TCS SP5 AOBS Tandem microscope and Leica Application Suite Advanced Fluorescence (LAS AF version 2.7.3.9723) software. Objectives LP/-/C HC PL APO 40x/1,30 OIL CS2 and LP/0,14–0,20/D HC PL APO 63x/1,40 OIL were used for imaging. In all cases, a single confocal z-stack is shown from one representative embryo of each genotype. For each marker, the number of positive cells and the total number of DAPI-positive (nuclei marker, blue) cells were enumerated using the cell counter plugin, FIJI software. The apoptotic index (cleaved caspase3), pro-apoptotic index (p53), proliferative index (Ki67) and mitotic index (phospho-H3-ser28) were calculated as the percentage of cells positive for each marker to the total number of DAPI-positive (nuclei marker, blue) cells in each of Epi, ExE and VE per embryo in a single confocal plane per embryo (at least 3 per group).

### RNA expression analysis and qPCR

After dissection of E6.25 *MommeD6* embryos and removal of their Reichert’s membrane, the epiblasts were carefully dissected from the rest of the embyo (ExE/EPC). Each isolated epiblast was lysed, snap-frozen at −80 °C, and were later extracted using an RNA micro kit (Qiagen) after the genotypes were determined by Sanger sequencing. Quality and concentration of eluted RNA was assessed with the Agilent RNA 6000 Pico Kit. Only samples with the RNA integrity score >8 were further processed for microarray analysis (Affymetrix GeneChip Mouse Gene 2.0 ST Array). For quantitative real time PCR (qPCR) total RNA from single epiblasts were isolated using a PicoPure RNA isolation kit (Life Technologies) and reactions were performed using the Roche LC480 light cycler. All qPCR primers are listed in Supplementary Table 2.

### Microarray analysis

Microarray data was processed from .CEL files using the *oligo* library in R^83^. Data was normalized using the RMA method and batch corrected using the *removeBatchEffects* function in *limma*^84^. Data was assessed pre and post normalization and corrected using the *prcomp* function and plotted to visualize sample in principal component space. Functional set enrichment was performed using SPIA^60^ and a modified version of *sigPathways*^85^ as described by Maciejewski^86^.

### Statistical analysis

All graphs were generated using GraphPad Prism version 7 and data are shown as mean and SEM. Mann-Whitney U test was used for analysing cell number; apoptotic, pro-apoptotic, proliferative, and mitotic indices and *p < 0.05 was considered significant.

### Data availability

Raw data is available from ArrayExpress (E-MTAB-5357)

## Electronic supplementary material

Supplementary Information File
