# Author Correction: The epigenetic modifier *Fam208a* is required to maintain epiblast cell fitness

**DOI:** 10.1038/s41598-018-22650-w

**Published:** 2018-04-05

**Authors:** Shohag Bhargava, Brian Cox, Christiana Polydorou, Veronika Gresakova, Vladimir Korinek, Hynek Strnad, Radislav Sedlacek, Trevor Allan Epp, Kallayanee Chawengsaksophak

**Affiliations:** 1 Laboratory of Transgenic Models of Diseases, Division, BIOCEV, Institute of Molecular Genetics of the CAS, v.v.i., Vestec, Czech Republic; 2 Czech Centre for Phenogenomics, Division BIOCEV, Institute of Molecular Genetics of the CAS, v.v.i., Vestec, Czech Republic; 3 Laboratory of Cell and Developmental Biology, Institute of Molecular Genetics of the CAS, v.v.i., Krc, Czech Republic; 4 Laboratory of Genomics and Bioinformatics, Institute of Molecular Genetics of the CAS, v.v.i., Krc, Czech Republic; 5 Department of Physiology, Faculty of Medicine, University of Toronto, Ontario, Canada; 6 Faculty of Science, Charles University, Prague, Czech Republic

Correction to: *Scientific Reports* 10.1038/s41598-017-09490-w, published online 24 August 2017

In the original version of this Article, an additional affiliation for Shohag Bhargava was omitted. The correct affiliations for Shohag Bhargava are given below:

Laboratory of Transgenic Models of Diseases, Division, BIOCEV, Institute of Molecular Genetics of the CAS, v.v.i., Vestec, Czech Republic.

Faculty of Science, Charles University, Prague, Czech Republic.

This error has now been corrected in the PDF and HTML versions of the Article, and in the Supplementary Information file.

