# Supplementary material for: The epigenetic modifier Fam208a is required to maintain epiblast cell fitness
Source: Sci Rep. 2017 Aug 24;7:9322. doi: 10.1038/s41598-017-09490-w (PMC5570896; doi:10.1038/s41598-017-09490-w)
Supplement: Supplementary file 1 — Supplementary Information File [file 41598_2017_9490_MOESM1_ESM.docx]

**Supplementary Information File**

The epigenetic modifier *Fam208a* is required to maintain epiblast cell potency

Shohag Bhargava^1,6^, Brian Cox^5^, Christiana Polydorou^1^, Veronika Gresakova^1^, Vladimir Korinek^3^, Hynek Strnad^4^, Radislav Sedlacek^1,2^, Trevor Allan Epp^1,2,*^ and Kallayanee Chawengsaksophak^1,2,*^

^1^Laboratory of Transgenic Models of Diseases, Division, BIOCEV, Institute of Molecular Genetics of the CAS, v.v.i., Vestec, Czech Republic

^2^Czech Centre for Phenogenomics, Division BIOCEV, Institute of Molecular Genetics of the CAS, v.v.i., Vestec, Czech Republic

^3^Laboratory of Cell and Developmental Biology, Institute of Molecular Genetics of the CAS, v.v.i., Krc, Czech Republic

^4^Laboratory of Genomics and Bioinformatics, Institute of Molecular Genetics of the CAS, v.v.i., Krc, Czech Republic

^5^Department of Physiology, Faculty of Medicine, University of Toronto, Ontario, Canada

^6^Faculty of Science, Charles University, Prague, Czech Republic

* Authors for correspondence:

**Kallayanee Chawengsaksophak, PhD & Trevor Allan Epp, PhD**

Institute of Molecular Genetics AS CR, v.v.i.

Videnska 1083, 142 20 Prague 4

Czech Republic

Tel.: +420 241 063 391

Fax: +420 224 310 955

**Email:** [kchaweng@img.cas.cz](mailto:kchaweng@img.cas.cz), [trevor.epp@img.cas.cz](mailto:trevor.epp@img.cas.cz)

**Running title:** *Fam208a* sustains epiblast potency

**Key words:** Momme, modifiers of murine metastable epialleles; PS, primitive streak; EMT, epithelial mesenchymal transition, gastrulation

# Supplementary Figure Legends


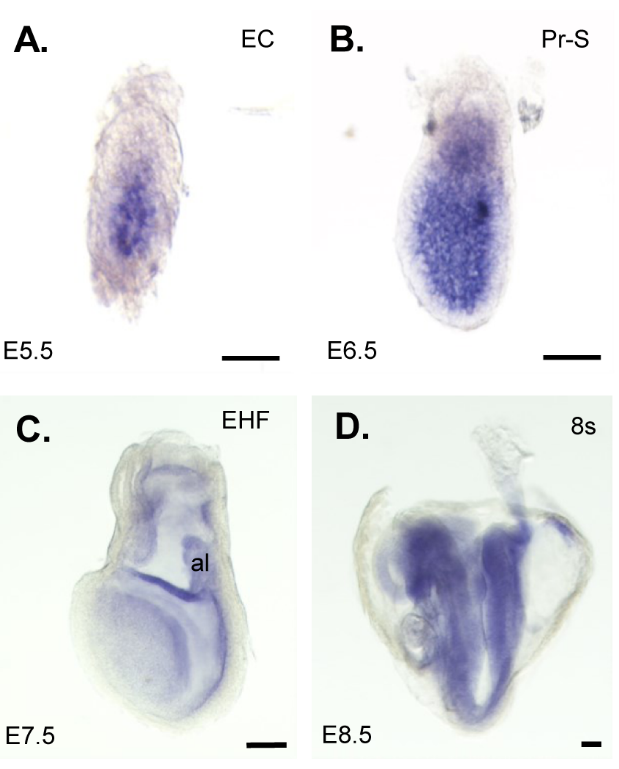


## Supplementary Fig. 1: *Fam208a* is widely expressed during early development.

Whole-mount in-situ hybridisation indicates widespread *Fam208a* expression at E5.5-7.5 (A - C) strongly seen in the epiblast and lesser in the ExE. Later, at E8.5-9.5, becomes strongly expressed ubiquitously (D - E). Scale bar: 100µm. EC, Egg Cylinder; Pr-S, Pre-streak; al, Allantois; EHF, Early Head Fold; 8s, 8 somites.


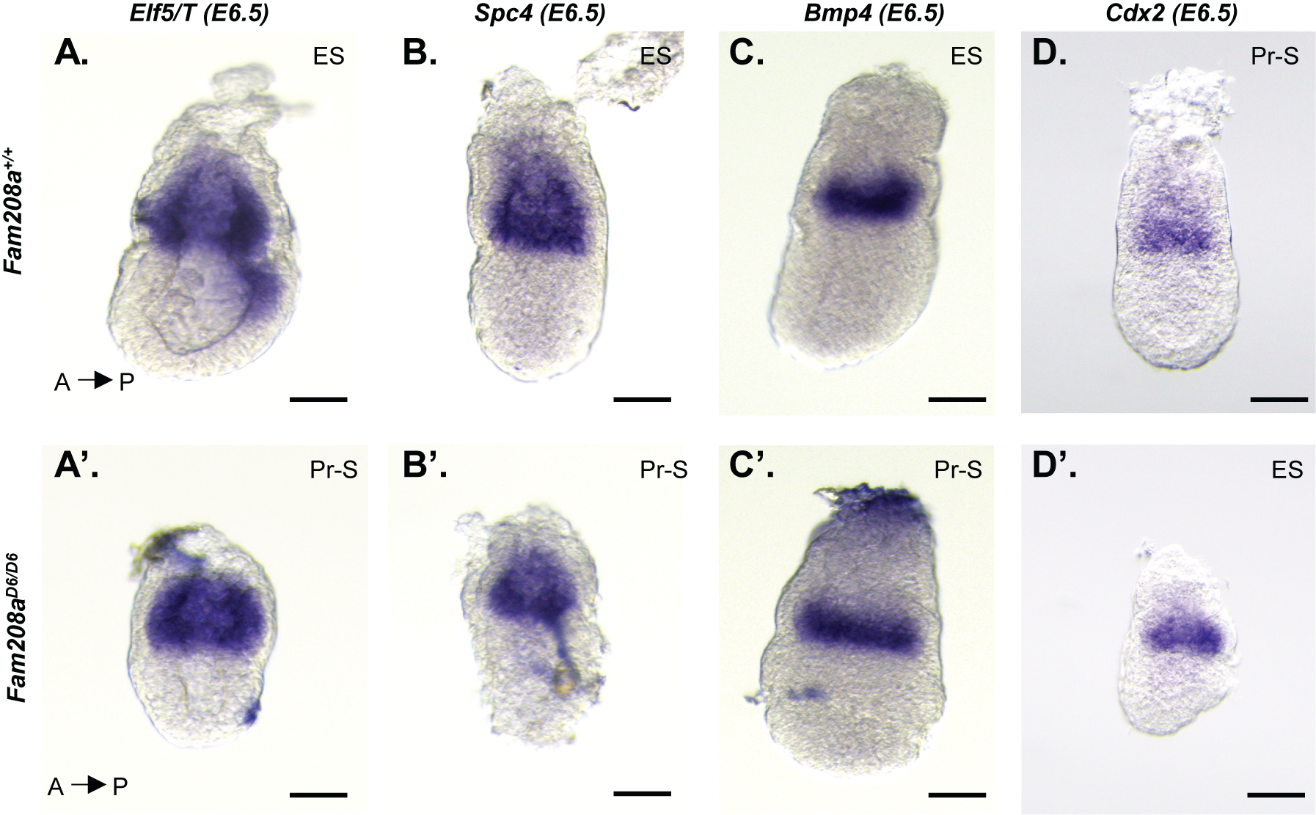


## Supplementary Fig. 2. Fam208aD6/D6 mutants exhibit minimal changes in extra-embryonic ectoderm (ExE) marker gene expression.

Whole mount in situ hybridisation at E6.5 of *Fam208a^D6/D6^* mutants (A’-D’) and their wild-type littermates (A-D) shows minimal expression changes of the ExE markers *Elf5*, (A’) *Spc4*, (B’) *Bmp4*, (C’) *Cdx2* (D’). Note that A-A’ show images from single-colored *in situ* hybridisation for the simultaneous detection of *Elf5* and *Brachyury (T).* Scale bar: 100µm. ES, Early streak; Pr-S, pre-streak. * indicates BM purple precipitate.

##
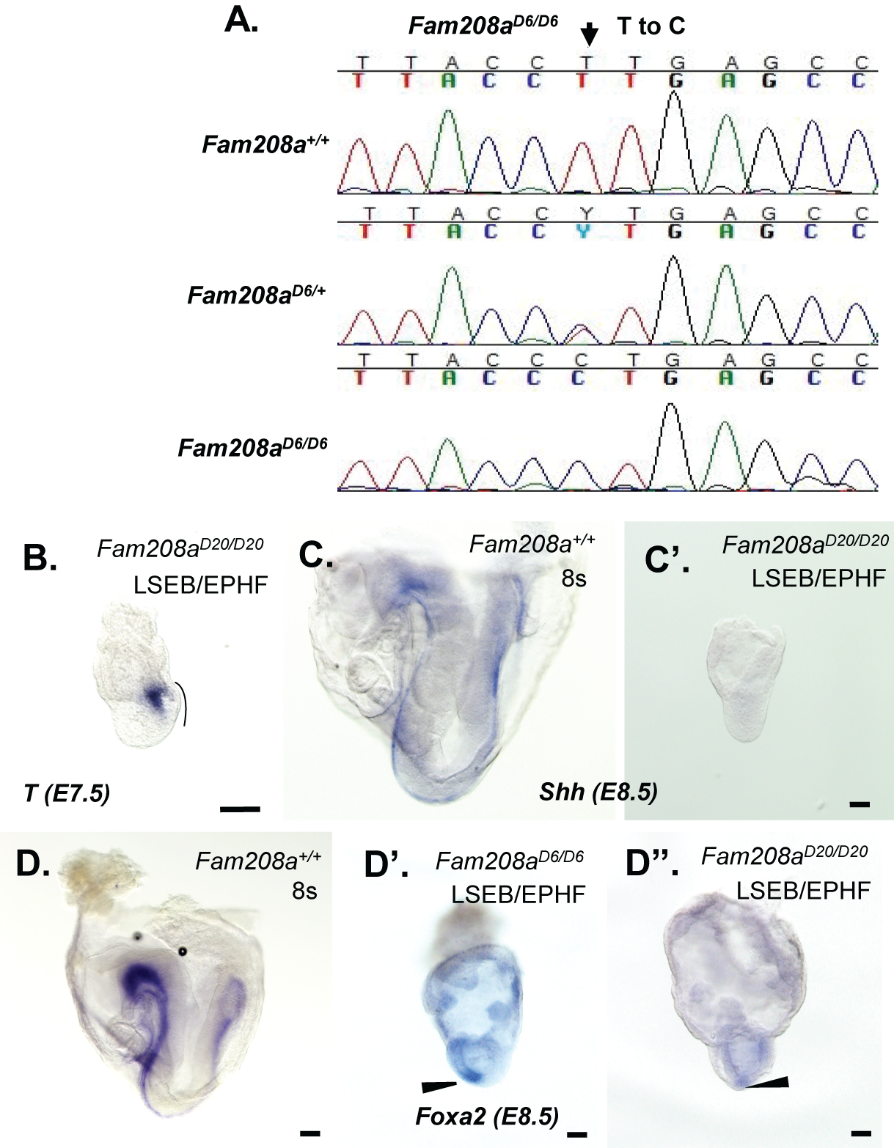


## Supplementary Fig. 3: *Fam208a* mutation leads to delayed AME derivatives at E8.5.

(A) Sanger sequence confirmation of *Fam208a^D6/D6^* mutation seen in the mutant embryos by genotyping. Whole mount in situ hybridisation at E7.5-E8.5 of *Fam208a* mutants (B, C’-D’& D’’) and their wild-type littermate controls (C & D) shows arrested expression of the primitive streak marker, *Brachyury*, (B) in the *Fam208a^D20/D20^* mutant embryos. (C’) Note that the mutants remain developmentally retarded with complete absence of *Shh* (Node &AME marker) expression. (D’-D’’) Reduced and distal expression of anterior mesendoderm (AME) marker *Foxa2* in seen in both *Fam208a* allelic mutants (indicated as arrowhead) with not much phenotypic variability. Scale bar: 100µm. EPHF, Early pre-head fold; LSEB, Late streak, EB, early allantoic bud; 8s, 8 somites; al, Allantois.


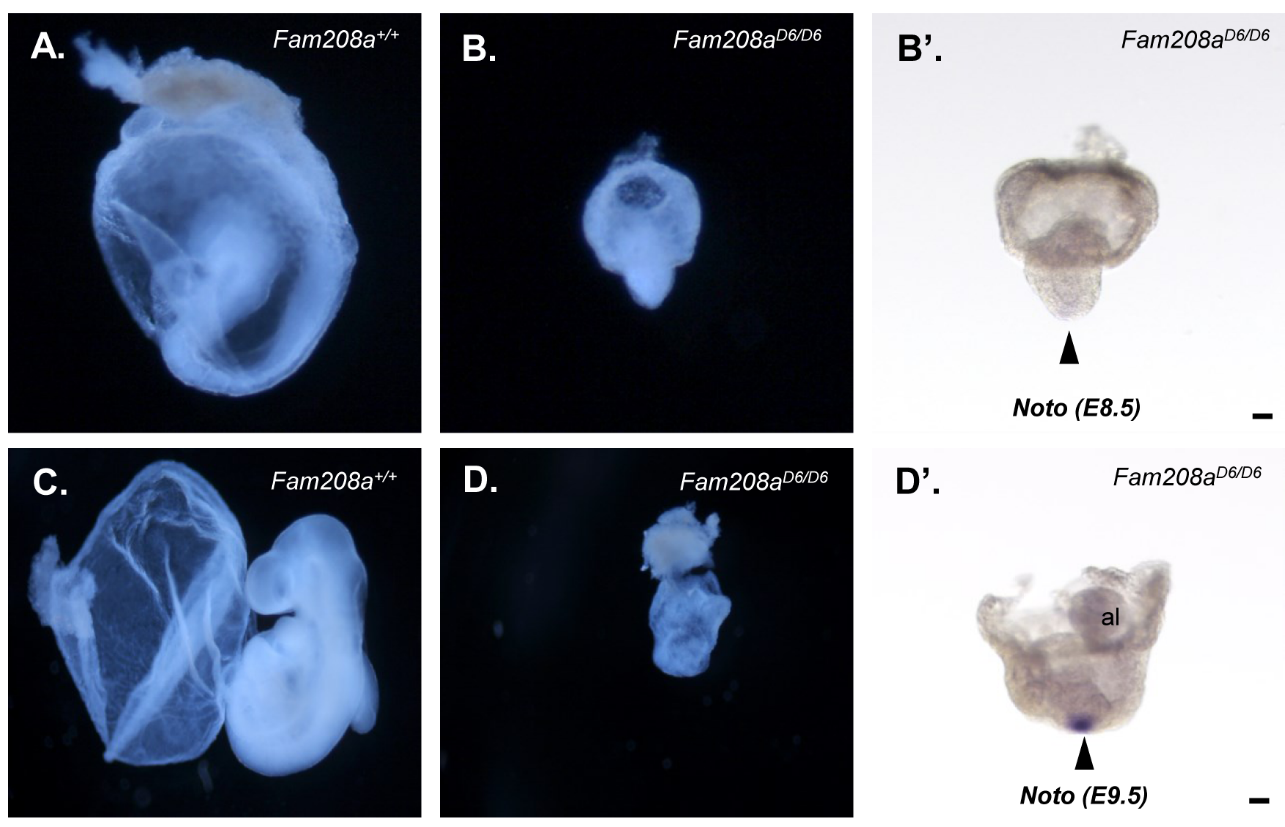


## Supplementary Fig. 4: *Fam208a* mutation leads to significant delay in the formation of node.

(A) Bright-field images of *Fam208a^D6/D6^* mutant embryos at E8.5-9.5 (B & D) and their wild-type littermate controls (A & C). Whole mount in situ hybridisation at E8.5-9.5 shows significantly delayed slight but correct distal expression of the node marker*, Noto*. Note, that the mutants also have the allantois. * asterisk indicates the node forming region.

Scale bar: 100µm. al, Allantois.


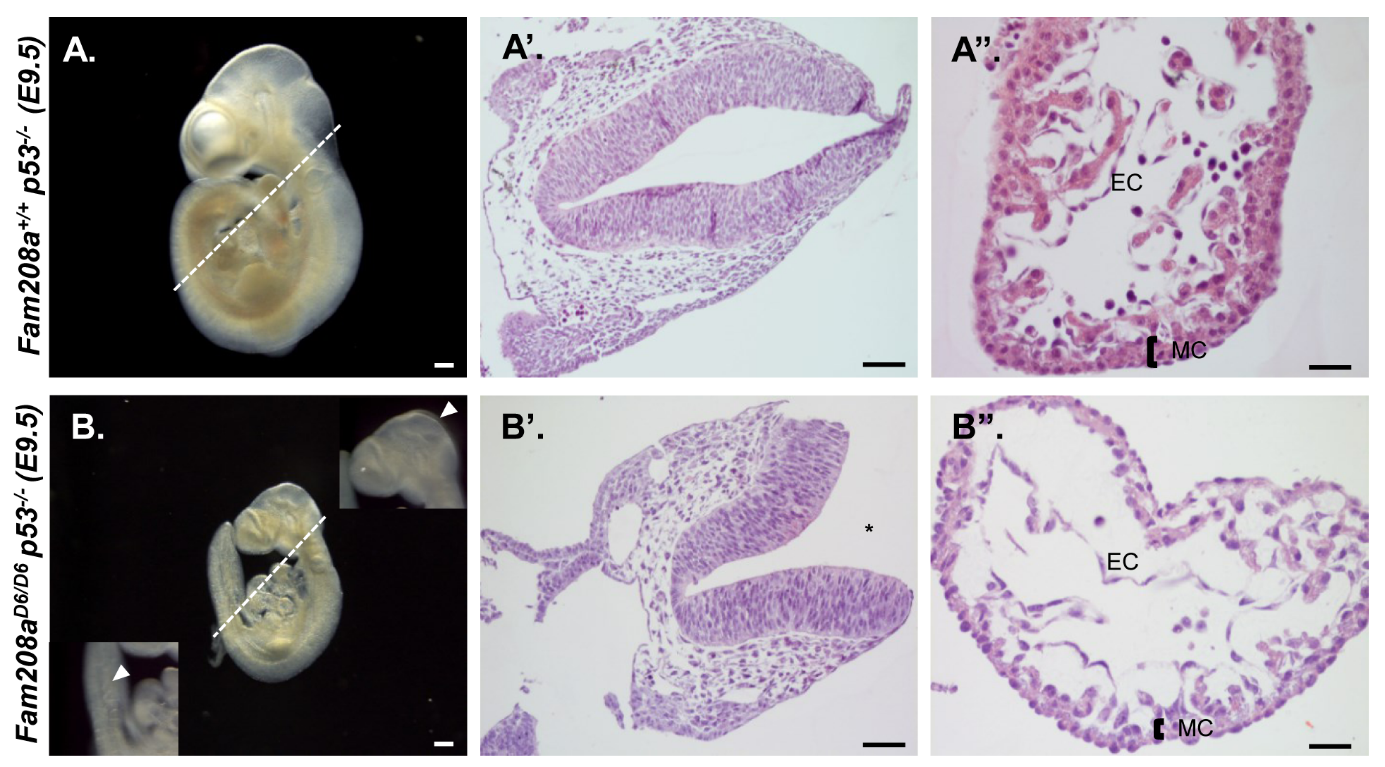


## Supplementary Fig. 5: Overt developmental defects highlighted in partially rescued *Fam208a^D6/D6^* embryos in a *p53^-/-^* background at E9.5.

(A-B) Bright-field images of mutant *Fam208a^D6/D6^* embryos, its corresponding rescued littermate at E9.5 (same embryo as represented in Fig. 8. E). Partial rescue of all homozygote *Fam208a* mutants in *p53^-/-^* background up to E8.5-9.0 with abnormalities in mid-hindbrain closure, kinky neural tube (arrowheads) and enlarged pericardium which can be seen in semi-thin transverse sections stained with Hematoxylin and Eosin (B’- B’’). The boxed region to the bottom left & right is of 3-fold magnification. Dotted line indicates the region of transverse-section. Arrowheads highlight developmental defects. Scale bar: 100µm.

##
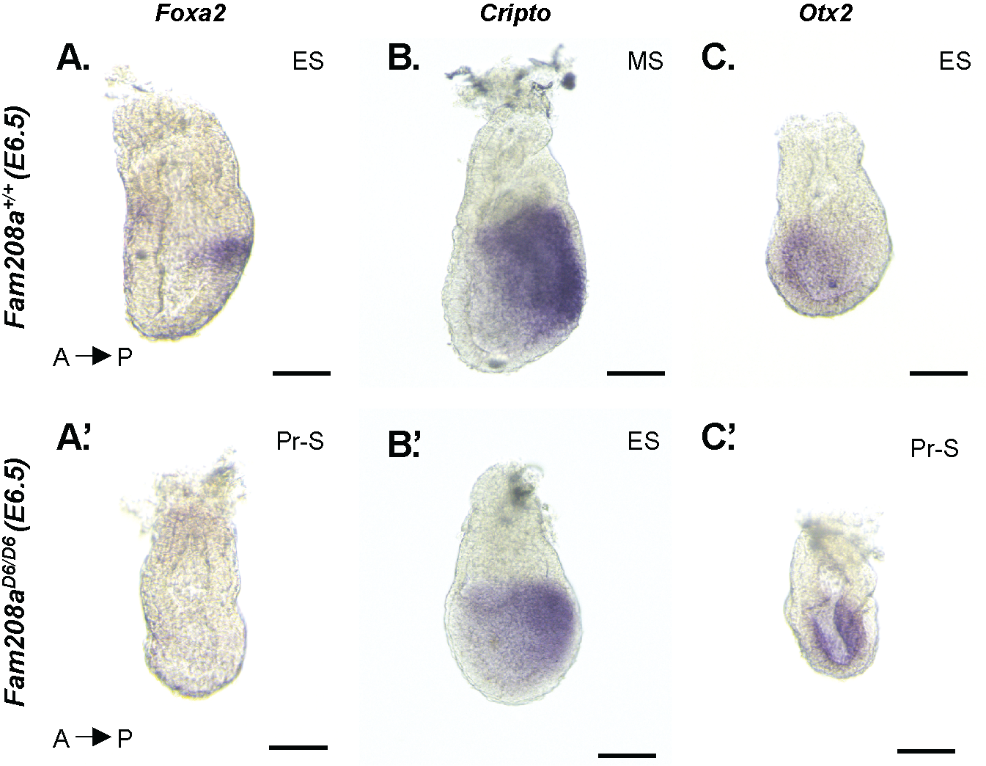


## Supplementary Fig. 6: Altered expression of epiblast specification markers in *Fam208a^D6/D6^* embryos at E6.5

Whole mount in situ hybridisation at E6.5 of *Fam208a^D6/D6^* mutants (A’-C’) and their wild-type littermates (A-C). The *Fam208a^D6/D6^* mutant embryos have no to faint Foxa2 expression (A’) with robust bilateral expression of Otx2 (B’). There is no discernible change in Cripto expression in the mutant embryo (C’). Scale bar: 100µm

# Supplementary Table S1. Selected functional gene set enrichment statistics using *sigPathways*.

## Homozygotes (*Fam208a^D6/D6^*)

| **Pathway_Name** | **Size** | **tstat.mean** | **Comp.Probability** | **NE_k_** | **NE_k_.p.val** | **NE_k_.q.val** |
| --- | --- | --- | --- | --- | --- | --- |
| p53/p73^a^ | 23 | 4.505 | <0.001 | 5.78 | 7.69E-09 | <0.001 |
| Platr^d^ | 20 | 6.389 | <0.001 | 3.09 | 2.00E-03 | 0.003 |
| KRAB^b^ | 132 | 0.479 | 0.001 | 1.71 | 8.70E-02 | 0.071 |
| Oct4coexpress^c^ | 423 | 2.390 | 0.005 | 2.75 | 6.00E-03 | 0.006 |

## Heterozygotes (*Fam208a*^+/^*^D6^*)

| **Pathway_Name** | **size** | **tstat.mean** | **Comp.Probability** | **NE_k_** | **NE_k_.p.val** | **NE_k_.q.val** |
| --- | --- | --- | --- | --- | --- | --- |
| p53/p73^a^ | 23 | 0.717 | 0.005 | 2.18 | 2.90E-02 | 0.094 |
| Platr^d^ | 20 | 0.590 | 0.044 | 1.36 | 1.74E-01 | 0.347 |
| Oct4coexpress^c^ | 423 | 0.138 | 0.242 | 0.60 | 5.51E-01 | 0.551 |
| KRAB^b^ | 132 | -0.009 | 0.957 | -0.16 | 8.70E-01 | 0.938 |

^a^the p53-bound (50kb from TSS) subset of genes significantly upregulated in response to combined p53/p73 depletion in mouse embryoid bodies (Wang et al, 2016).

^b^gene set defined according to InterPro ID (IPR001909: Krueppel-associated box).

^c^pluripotency-associated Oct4 co-expression module (Bergmann et al, 2015).

^d^pluripotency-associated long non-coding transcripts (Bergmann et al, 2015) .

# Supplementary Table S1. qPCR primers

|  |  |
| --- | --- |
| **Oligo Name** | **Sequence** |
|  |  |
| mCdkn1a-F | AACATCTCAGGGCCGAAA |
| mCdkn1a-R | TGCGCTTGGAGTGATAGAAA |
| mCcng1-F | TGGACAGATTCTTGTCTAAAATGAAG |
| mCcng1-R | CAGTGGGACATTCCTTTCCTC |
| mDkk1-F | CCGGGAACTACTGCAAAAAT |
| mDkk1-R | CCAAGGTTTTCAATGATGCTT |
| mWnt3-F | GATGTGGAGGCAGGTCTCTT |
| mWnt3-R | CAGAGCAGCCCATTCTTTCT |
| mEomes-F | AGCAGCCCAGAGGGTTAAA |
| mEomes-R | TGAAGAGCCCACTGTTAACTCA |
| mOct4-F | AATGCCGTGAAGTTGGAGAA |
| mOct4-R | CCTTCTGCAGGGCTTTCAT |
| mPlatr4-F | TGTGAGAATCAGGGAAAGTGG |
| mPlatr4-R | TGAGTGCTGAGTTGCAGGTT |
| mPlatr20-F | CGGGAAAGCAGAGTGCTG |
| mPlatr20-R | TTGCCTTGTTTTTCAAATAGTACCT |
| mPlatr27-F | GACTCAGCTGGGTTCCAGAG |
| mPlatr27-R | CTGGCTCTTCAAGTCTTCTGC |
| mGAPDH | GGGTTCCTATAAATACGGACTGC |
| mGAPDH | CCATTTTGTCTACGGGACGA |

All real-time PCR primer pairs were designed with Tm approximately 60 ⁰C using the Universal Probe Library Tool on the Roche Website (https://lifescience.roche.com/en_cz/brands/universal-probe-library.html#assay-design-center)

# Supplementary Table S3. List of primary and secondary antibodies

**Primary antibodies**

| Antibody | Cat. No. | Dilution |
| --- | --- | --- |
| anti-Ki67 | Abcam #ab15580 | 1:250 |
| anti-phospho-Histone H3 (Ser28) | Cell Signaling # 9713 | 1:250 |
| anti-Cleaved Caspase3 | Cell Signaling #9664 | 1:250 |
| anti-p53 | Novocastra #NCL-p53-CM5p | 1:250 |
| anti-Oct4 | Santa Cruz #sc-5279 | 1:100 |
| anti-Snail | Cell Signaling # C15D3 | 1:250 |
| anti-E-Cadherin | Sigma Aldrich #U3254 | 1:500 |

**Secondary antibodies**

| Antibody | Cat. No. | Dilution |
| --- | --- | --- |
| Alexa Flour 488-conjugated goat anti-rabbit | Invitrogen #A-11034 | 1:500 |
| Alexa Flour 594-conjugated goat anti-rabbit | Invitrogen # A-11012 | 1:500 |
| Alexa Flour 594-conjugated goat anti-rat | Invitrogen # A-11007 | 1:500 |
| Alexa Flour 488-conjugated goat anti-mouse | Jackson# 715-545-150 | 1:500 |
